# Diel hysteresis between soil respiration and soil temperature in a biological soil crust covered desert ecosystem

**DOI:** 10.1371/journal.pone.0195606

**Published:** 2018-04-06

**Authors:** Chao Guan, Xinrong Li, Peng Zhang, Yongle Chen

**Affiliations:** 1 Shapotou Desert Research and Experimental Station, Northwest Institute of Eco-Environment and Resources, Chinese Academy of Sciences, Lanzhou, Gansu, China; 2 University of Chinese Academy of Sciences, Beijing, China; Pacific Northwest National Laboratory, UNITED STATES

## Abstract

Soil respiration induced by biological soil crusts (BSCs) is an important process in the carbon (C) cycle in arid and semi-arid ecosystems, where vascular plants are restricted by the harsh environment, particularly the limited soil moisture. However, the interaction between temperature and soil respiration remains uncertain because of the number of factors that control soil respiration, including temperature and soil moisture, especially in BSC-dominated areas. In this study, the soil respiration in moss-dominated crusts and lichen-dominated crusts was continuously measured using an automated soil respiration system over a one-year period from November 2015 to October 2016 in the Shapotou region of the Tengger Desert, northern China. The results indicated that over daily cycles, the half-hourly soil respiration rates in both types of BSC-covered areas were commonly related to the soil temperature. The observed diel hysteresis between the half-hourly soil respiration rates and soil temperature in the BSC-covered areas was limited by nonlinearity loops with semielliptical shapes, and soil temperature often peaked later than the half-hourly soil respiration rates in the BSC-covered areas. The average lag times between the half-hourly soil respiration rates and soil temperature for both types of BSC-covered areas were two hours over the diel cycles, and they were negatively and linearly related to the volumetric soil water content. Our results highlight the diel hysteresis phenomenon that occurs between soil respiration rates and soil temperatures in BSC-covered areas and the negative response of this phenomenon to soil moisture, which may influence total C budget evaluations. Therefore, the interactive effects of soil temperature and moisture on soil respiration in BSC-covered areas should be considered in global carbon cycle models of desert ecosystems.

## Introduction

Soil respiration accounts for the largest proportion of the total ecosystem respiration [1], and its global integration is an order of magnitude larger than that of anthropogenic CO_2_ releases from burning fossil fuels and deforestation [1,2]. However, soil respiration processes are not well documented in arid and semi-arid ecosystems, which cover approximately 45% of the global land surface and contain 20% of the global soil C pool [3,4]. Soil respiration processes in arid and semi-arid ecosystems are different from those in other ecosystems because of the effects of drought and high temperatures [3,5]. Thus, a further understanding of soil respiration in arid and semi-arid ecosystems is necessary to ensure accurate representation in large-scale carbon models.

Biological soil crusts (BSCs) are widespread communities that consist of cyanobacteria, green algae, lichens, mosses and other organisms, and they are closely integrated with particles of the soil surface in arid and semi-arid regions [6]. These communities have long been acknowledged as one of the major components of arid and semi-arid ecosystems [7,8], where up to 70% of the living ground is covered by BSCs in certain plant communities [9]. BSCs play an important role in ensuring the proper structure and function of desert ecosystems, by alleviating soil erosion [6,10], fixing carbon (C) and nitrogen [11,12], and creating favorable microhabitats for plants and soil arthropods [6,8]. BSCs have been suggested as one of the critical factors responsible for the large annual CO_2_ net uptake rates recorded in desert ecosystems [13]. Previous research found that BSC-dominated microsites accounted for 42% of the total C emitted through soil respiration, whereas vegetated and bare soil accounted for 37% and 20%, respectively, in a semi-arid ecosystem [14]. Another work showed that algal crusts and subsurface microbial respiration accounted for approximately 60% of the total C release in the soil during the growing season [15]. Therefore, BSCs need to be considered when estimating the C budgets in desert ecosystems because of their major contribution to the total C release by soil respiration.

Automated soil respiration systems have been widely used to provide continuously dense datasets that reveal a complex relationship between soil respiration and temperature, which is not easily illustrated using less frequent survey measures. Numerous studies have analyzed the data from automated soil respiration systems and found significant diel hysteresis, which is supported by the semielliptical shapes observed in the regression analyses between temperature and soil respiration [16–19]. Studies have indicated that low soil moisture increases the degree of diel hysteresis between soil respiration and temperature [17,19,20], whereas the opposite results have also been reported [16]. Both biological and physical explanations have been proposed for the hysteresis patterns [17]. However, a consensus has not been reached on the causes of the diel hysteresis patterns, especially in BSC-dominated areas in desert ecosystems with sandy soil and low soil moisture. The specific objectives of this study were (1) to explore the seasonal relationships between the diurnal cycles of soil respiration and soil temperature in BSC-dominated ecosystems and (2) evaluate the effects of soil moisture on diel hysteresis in the relationships between soil respiration and soil temperature in BSC-dominated soils over the seasonal cycles.

## Materials and methods

### Ethics statement

This experiment was conducted at the Shapotou Desert Research and Experimental Station, Chinese Academy of Sciences (37°27′ N, 105°07′ E), a department of the Northwest Institute of Eco-Environment and Resources, Chinese Academy of Science. The study was approved by the Northwest Institute of Eco-Environment and Resources, Chinese Academy of Sciences. No specific permissions were required for sample collection in the Shapotou-Yiwanquan region (37°25′ N, 104°40′ E), and the field work did not involve any endangered or protected species.

### Study area

This study was conducted in the Shapotou-Yiwanquan region (37°25′ N and 104°40′ E, 1339 m AMSL), which is located in Zhongwei County in the Ningxia Hui Autonomous Region at the southeastern edge of the Tengger Desert in northern China. This area is a transitional zone between desertified steppe and sandy desert [21]. The mean annual air temperature is 9.6°C, and the mean monthly temperature is 24.3°C in July and -6.9°C in January. The mean annual precipitation is 186 mm, and 80% is received between May and September. The potential evapotranspiration is approximately 2900 mm during the growing season (April to October). The annual mean wind velocity is 2.6 m/s, and the windy season is from September to April. The annual mean number of dust-storm days is 59.

A field investigation of the vegetation was conducted in July 2015. The dominant natural shrub species are *Caragana korshinskii* and *Artemisia ordosica*, and the total plant cover is approximately 38.6%. The predominant natural herbal species are *Echinops gmelinii*, *Artemisia capillaries*, *Allium mongolicum* and *Salsola ruthenica*, which present coverage of approximately 29.1%. In addition, open areas between plant patches, which consist of moss-dominated and lichen-dominated crusts, have a cover of approximately 51.2% and 33.6%, respectively. The moss species include *Didymodon constrictus* (Mitt.) Saito., *Bryum argenteum* Hedw., *Tortula desertorum* Broth., and *Tortula bidentata* Bai Xue Liang [22]. The lichen species include *Endocarpon pusillum* Hedw., *Collema tenax* (Sw.) Ach., and a number of *Diploschistes muscorum* (Scop.) R. Sant and *Squamarina lentigera* (G.H. Weber) Poelt [23]. The physical and chemical characteristics of the two BSC-dominated soils at a depth of 0–5 cm are provided in Table 1.

**Table 1:** Physical and chemical characteristics of the BSC-dominated soils at a depth of 0–5 cm.

Soil properties	Crust type	
	moss-dominated crusts	lichen-dominated crusts
**pH value**	8.65 ± 0.05	8.67 ± 0.03
**Organic matter (%)**	1.27 ± 0.06	1.09 ± 0.05
**Total nitrogen (%)**	0.033 ± 0.003	0.015 ± 0.003
**Total phosphorus (%)**	0.054 ± 0.003	0.045 ± 0.002
**C/N ratio**	22.79 ± 0.76	44.36 ± 6.10
**Sand (%)**	75.11 ± 4.01	84.67 ± 3.45
**Silt (%)**	14.11 ± 2.33	9.32 ± 2.11
**Clay (%)**	10.78 ± 1.71	6.01 ± 1.41

### Experimental design

In July 2015, intact BSC samples (moss-dominated crusts and lichen-dominated crusts) were collected using PVC collars (20 cm in diameter and 20 cm in height) in the Shapotou-Yiwanquan region. To avoid terrain and vegetation influences on BSC development, all samples were randomly collected from undisturbed soil in the spaces between shrubs. The BSC samples were taken to the Shapotou Desert Research and Experimental Station and randomly buried in the soil (still in the PVC collars) of six plots (10 m × 10 m each), and the BSC surfaces were placed at the same level as the soil surface. To ensure that the test conditions closely resembled the natural environment, we maintained the natural soil water and air cycles by keeping the bottoms of the PVC collars open for drainage. Each type of BSC had three replicates in each plot, and they were incubated for four months before the first measurements.

### Soil respiration measurements

The soil respiration rates of the BSC (R_BSC_, μmol m^-2^ s^-1^) were measured using an Automated Soil Respiration System (LI-8100A fitted with a LI-8150 multiplexer, LI-COR Inc., Lincoln, Nebraska, USA) from November 2015 to October 2016. A permanent opaque chamber (LI-104, LI-COR Inc., Lincoln, Nebraska, USA) was set on each collar. The measurement time for each chamber was 3 min and 10 s, including a 45 s pre-purge, a 45 s post-purge, a 90 s observation period, and a 10 s dead band. During the measurements, one-third of the samples were randomly measured on the first day, and the other samples were measured on the following two days. All plant seedlings present within the measurement collar were manually removed. The half-hourly soil temperature (T) and volumetric soil water content (VWC) were measured at a 5 cm soil depth outside of each chamber using the 8150–201 soil temperature sensor and 8150–204 ML2x soil moisture sensor (LI-COR Inc., Lincoln, Nebraska, USA). Rainfall was measured at a distance of 1.6 km from the research location using a manual rain gauge during the experiment.

### Data analysis

All R_BSC_ data were screened using limit checking. Half-hourly CO_2_ effluxes less than -0.5 were considered abnormal and removed from the data set [19]. Instrument failure (from 24 February 2016 to 23 March 2016) and the quality control procedures resulted in 11% missing data during the measurements. The mean diel cycles of R_BSC_ and temperature for each month were calculated as the average of the half-hourly means for each time of day, and the cycles were then used to analyze the diel variation in R_BSC_ and identify the hysteresis between R_BSC_ and temperature. A cross-correlation analysis was used to detect the hysteresis between R_BSC_ and temperature in the diel cycles, and synchronize the values before the regression was performed [24,25]. A regression analysis was used to evaluate the relationships between R_BSC_ and temperature, the hysteresis in the R_BSC_-temperature relationship and the VWC. All of the analyses were performed using SPSS 16.0 statistical software (SPSS Inc., Chicago, IL, USA).

## Results

### Seasonal variation in environmental factors and soil respiration in BSC-covered areas

**Fig 1:**
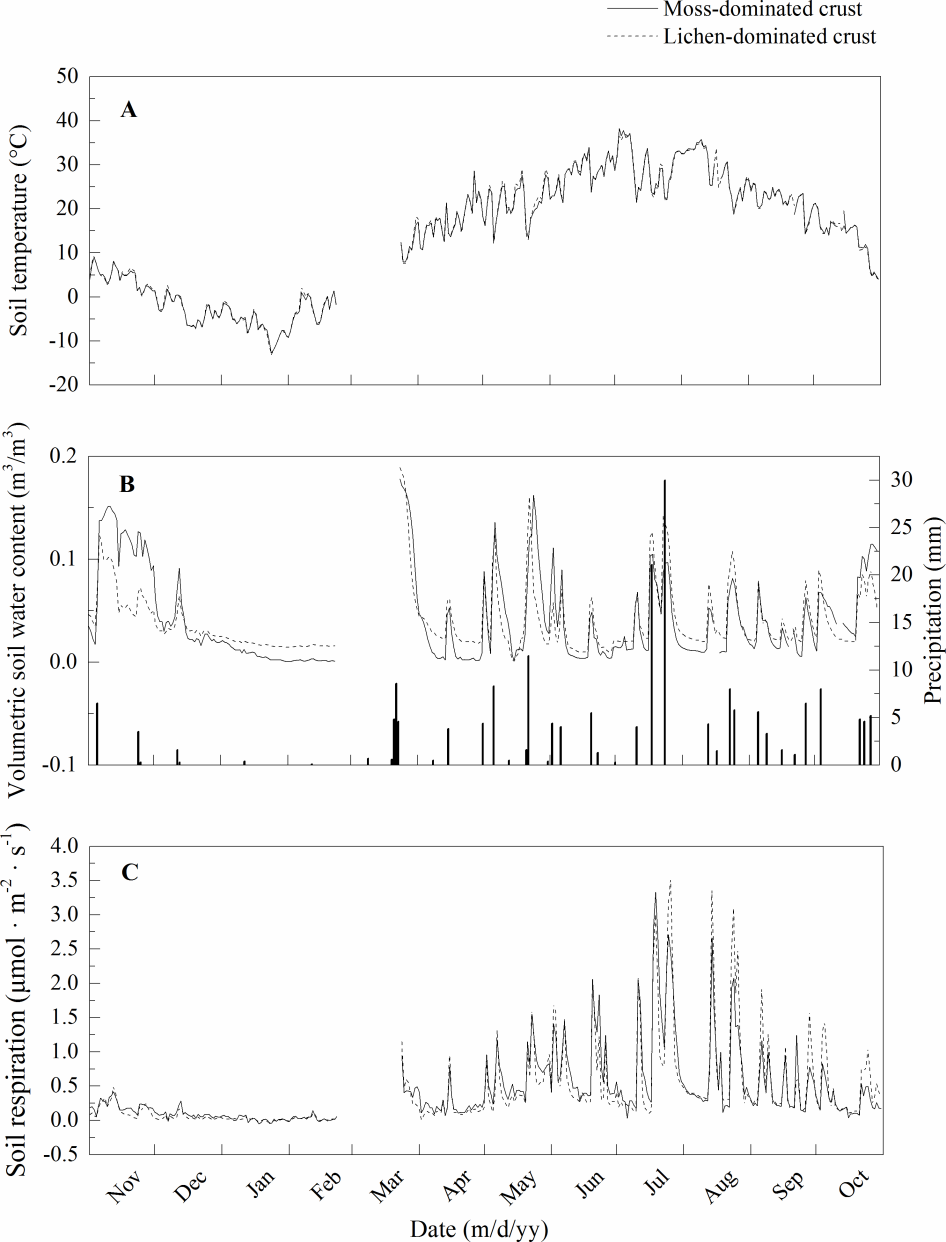
**Daily mean soil temperature at a depth of 5 cm (A), volumetric soil water content at a depth of 5 cm (B), and soil respiration (C) in the BSC-covered areas during the experimental periods from November 2015 to October 2016 in the Shapotou region of the Tengger Desert, northern China.** Error bars of the soil temperature are omitted for clarity (n = 48).

Similar changes were observed in the soil respiration as well as the daily mean soil temperature and VWC at a depth of 5 cm in both the moss-dominated crusts and lichen-dominated crusts (Fig 1). The minimum daily mean soil temperature at a depth of 5 cm in the moss-dominated crusts was -12.6°C in January and reached a maximum value of 38.2°C in July (Fig 1A). For the lichen-dominated crusts, the minimum daily mean soil temperature at a depth of 5 cm was -13.1°C in January and reached a maximum value of 37.0°C in July (Fig 1A). The total precipitation from November 2015 to October 2016 was 193.9 mm (Fig 1B), which was close to the average record of 186.2 mm. During the measurement period, 68.3% of rainy days had rainfall amounts of 5 mm or less (Fig 1B), and the distribution pattern matched the 30 years of precipitation records from our study site in the Shapotou region in China. The daily mean VWC at a depth of 5 cm ranged from 0.001 to 0.178 m^3^/m^3^ in the moss-dominated crusts and from 0.001 to 0.189 m^3^/m^3^ in the lichen-dominated crusts (Fig 1B). The daily mean soil respiration rates in the moss-dominated crusts and lichen-dominated crusts varied markedly following changes in soil temperature and VWC at a depth of 5 cm, especially after a rain pulse. The minimum daily mean soil respiration rates in the moss-dominated crusts was -0.052 μmol m^-2^ s^-1^ in January, reaching a maximum value of 3.329 μmol m^-2^ s^-1^ in July (Fig 1C, S1 Table). For the lichen-dominated crusts, the minimum daily mean soil respiration rates were -0.032 μmol m^-2^ s^-1^ in January, and reached a maximum value of 3.514 μmol m^-2^ s^-1^ in July (Fig 1C, S1 Table).

### Diel hysteresis between soil respiration and soil temperature in BSC-covered areas and its response to soil moisture

**Fig 2:**
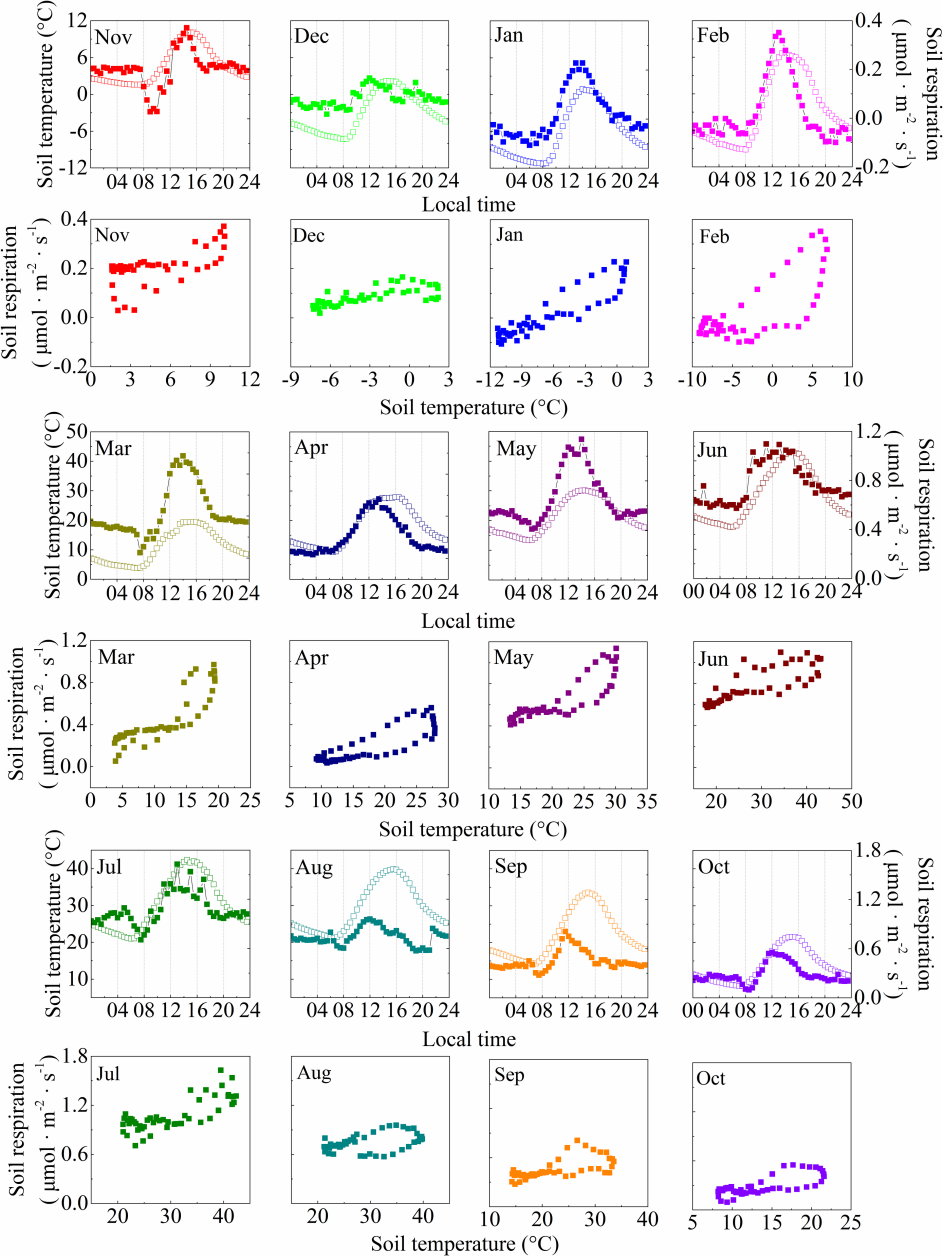
Mean monthly diel cycles of soil respiration (solid points) and soil temperature at a depth of 5 cm (open points) in the moss-dominated crusts. Each point is the monthly mean for a particular time of day.

**Fig 3:**
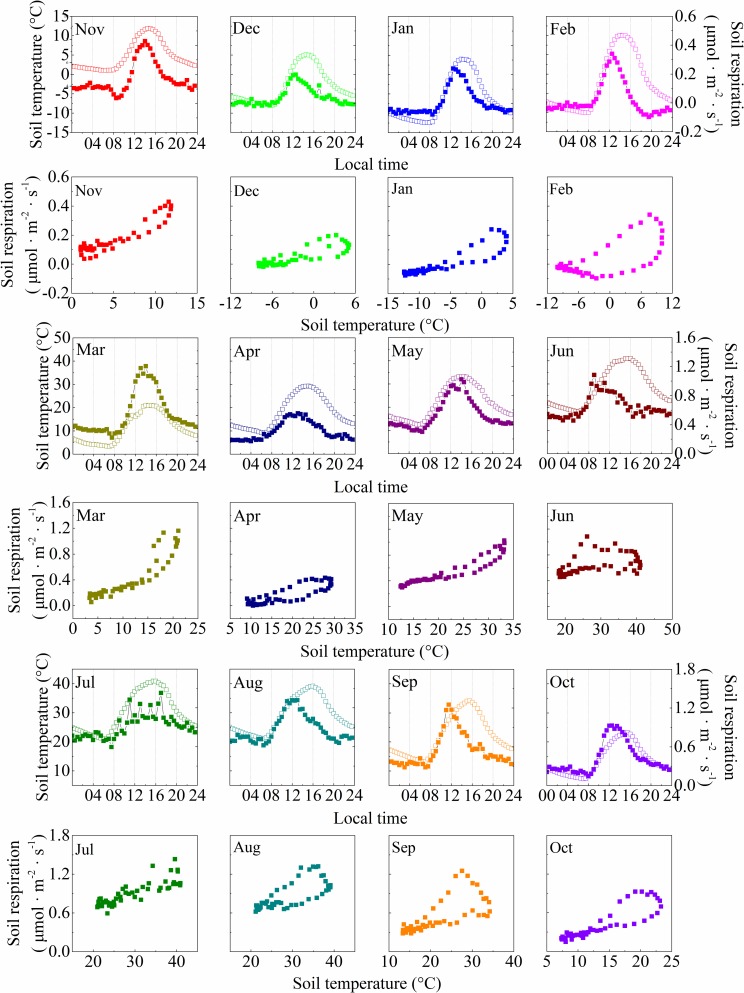
Mean monthly diel cycles of soil respiration (solid points) and soil temperature at a depth of 5 cm (open points) in the lichen-dominated crusts. Each point is the monthly mean for a particular time of day.

**Fig 4:**
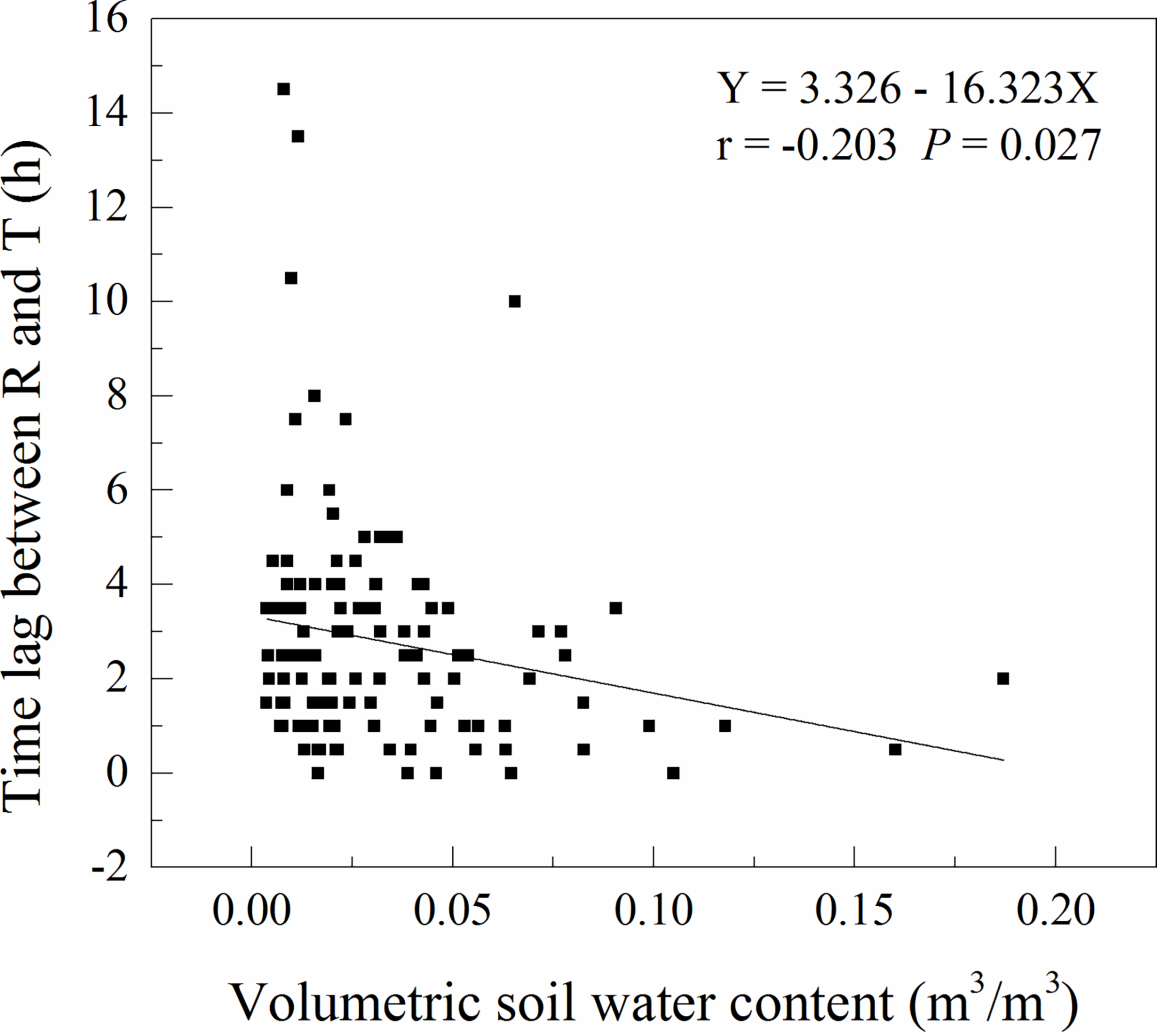
Lag times between soil respiration and soil temperature at a depth of 5 cm in the BSC-covered areas over the diel cycles, in relation to the volumetric soil water content. The lag times were calculated from the data in five-day intervals.

Because of the marked seasonal variations in soil temperature and moisture, the half-hourly soil respiration rates in the moss-dominated crusts and lichen-dominated crusts presented a temporal variation from November 2015 to October 2016. The relationship between the half-hourly soil respiration rates and soil temperature at a depth of 5 cm in the two types of BSC-covered areas showed daily hysteresis loops with semielliptical shapes each month over the daily cycles, and the hysteresis loops varied at a temporal scale among months (Figs 2 and 3). Over the daily cycles, the half-hourly soil respiration rates in both types of BSC-covered areas were out of phase with the soil temperature at a depth of 5 cm throughout the entire sampling year, which is consistent with the diel hysteresis observed in the relationship between the half-hourly soil respiration rates and the soil temperature in both types of BSC-covered areas (Figs 2 and 3). The maximum half-hourly soil respiration rates in the moss-dominated crusts occurred between 11:00 LT (GMT + 8) and 14:30, and the maximum soil temperature at a depth of 5 cm occurred between 14:00 and 16:00 (Fig 2). For the lichen-dominated crusts, the maximum half-hourly soil respiration rates occurred between 9:00 and 17:00, and the maximum soil temperature at a depth of 5 cm occurred between 14:00 and 16:00 (Fig 3). The mean diel cycles of the half-hourly soil respiration rates and the soil temperature at a depth of 5 cm in the BSC-covered areas showed significant positive correlations for all months during the experiment. The lag times between the half-hourly soil respiration rates and soil temperature at a depth of 5 cm in the BSC-covered areas over the daily cycles were two hours on average (Table 2), and the soil respiration rates peaked earlier than the soil temperature at a depth of 5 cm in the BSC-covered areas. The time lags between the half-hourly soil respiration rates and soil temperature at a depth of 5 cm in the BSC-covered areas were negatively and linearly related to the VWC (Fig 4; Lag (h) = 3.326–16.323 VWC, r = -0.203, *p* = 0.027).

**Table 2:** Analysis of the mean monthly diel cycles of soil respiration and soil temperature at a depth of 5 cm in the BSC-covered areas, including the correlation coefficients before synchronization and lag times in the diel cycles. The Pearson correlation coefficients and *p* values are given.

Time	Moss-dominated crust			Lichen-dominated crust		
	Pearson’s r	Lag time	*p* value	Pearson’s r	Lag time	*p* value
**Nov 2015**	0.600	0.5 h	<0.001	0.919	0.5 h	<0.001
**Dec 2015**	0.713	3.5 h	<0.001	0.838	2 h	<0.001
**Jun 2016**	0.911	1 h	<0.001	0.899	1.5 h	<0.001
**Feb 2016**	0.746	1 h	<0.001	0.679	1.5 h	<0.001
**Mar 2016**	0.864	2 h	<0.001	0.911	0 h	<0.001
**Apr 2016**	0.840	2.5 h	<0.001	0.849	2 h	<0.001
**May 2016**	0.852	0.5 h	<0.001	0.935	0.5 h	<0.001
**Jun 2016**	0.814	4 h	<0.001	0.427	4 h	0.002
**Jul 2016**	0.770	1.5 h	<0.001	0.837	-1.5 h	<0.001
**Aug 2016**	0.424	3.5 h	0.003	0.723	3 h	<0.001
**Sep 2016**	0.644	3.5 h	<0.001	0.708	4 h	<0.001
**Oct 2016**	0.733	3.5 h	<0.001	0.876	3 h	<0.001

## Discussion

Continuous measurements of half-hourly soil respiration rates in both the moss-dominated and lichen-dominated crusts were conducted with an automated soil respiration system in a desert ecosystem and provide new insights into the control of soil respiration in BSC-covered areas base on environmental variables at diurnal and seasonal time scales. Over the daily cycles, the half-hourly soil respiration rates in both types of the BSC-covered areas were commonly related to soil temperature, and soil temperature often peaked later than the half-hourly soil respiration rates in the BSC-covered areas.

### Diel hysteresis between soil respiration and soil temperature in BSC-covered areas

Over the course of the diurnal cycles, our results demonstrate that a significant time lag occurred between the half-hourly soil respiration rates and soil temperature at a depth of 5 cm in both types of BSC-covered areas, with the soil respiration peaking earlier than the soil temperature (Figs 2 and 3). A similar phenomenon has also been observed in desert ecosystems [19,26,27]. However, studies on an oak-grass savanna [28], boreal aspen stand [29], mixed conifer and oak forest [30], and wheat field [31] found an opposite time lag between the soil respiration rates and the temperature, with soil respiration peaking later than the temperature. Although the reason for the time lag is still unclear, several mechanisms have been proposed to explain the causes of the diel hysteresis. First, the photosynthetic C supply presents diurnal variations and fluctuates out of phase with the soil temperature [28,30,32,33]. Second, autotrophic respiration is affected by photosynthetically active radiation [34] and air temperatures, whereas heterotrophic respiration is primarily affected by soil temperature [35,36], and the different responses of autotrophic respiration and heterotrophic respiration to environmental factors may explain the observed diel hysteresis [16]. Third, recent research has shown that more isotopically depleted values occurred at night along with the diel variation in respiratory ^13^CO_2_ values, and most of the depletion was observed on nights when the atmospheric CO_2_ showed strong increases [37]. This observed diurnal variation in respiratory ^13^CO_2_ values was hypothesized to be the result of the non-steady state process “diffusive fractionation”, which is induced by the increasing atmospheric CO_2_ concentrations at nighttime, especially in arid ecosystems [37]. Lastly (and perhaps most importantly), the CO_2_ efflux is strongly coupled with the temperature and moisture of the surface BSC layer in the BSC interspaces, whereas it is less strongly correlated with the temperature and moisture of the deeper soil layers [38]. In addition, BSC represent the major component of autotrophic respiring biomass in BSC-dominated areas [14,15,39]. Thus, the proximate cause of the diel hysteresis in our study is likely a mismatch between the depth of the soil temperature and moisture measurements and the depth of CO_2_ production [40–42]. Thus, to further investigate the mechanisms underlying the diel hysteresis between soil respiration and soil temperature in BSC-covered areas, additional modeling and controlled experiments are needed.

### Changes in diel hysteresis between soil respiration and soil temperature in BSC-covered areas under differences in soil moisture

Soil respiration and soil temperature present a time lag, and these variables are plotted against each other in a semielliptical form, and vary seasonally with the soil moisture [16, 19, 28]. In our study, the mean lag times between the half-hourly soil respiration rates and soil temperature at a depth of 5 cm in the two types of BSC-covered areas were on average two hours over the diel cycle (Table 2), and the lag times were negatively and linearly related to the soil moisture (Fig 4). Our results are supported by Feng et al. [27], who also found that the lag times between the soil temperature at a depth of 5 cm and soil respiration were negatively correlated with the VWC in a crusted desert ecosystem. Similarly, a study in a desert shrub ecosystem revealed that the lag times between the soil temperature at a depth of 10 cm and soil respiration increased as the VWC decreased [19], and the authors attributed the increased lag times under decreased VWC to the decoupling of soil respiration from soil temperature under low soil water contents in desert ecosystems [19,27]. A study in a semi-arid desert showed that the magnitude of the diel hysteresis between soil respiration and soil temperature at a depth of 10 cm was modified by precipitation, with the lag time decreasing after rainfall when the soil is wetted up [26]. The decreases lag time with increased VWC is likely because water transfers heat more efficiently than air; thus, when the soil profile is saturated with water (high VWC), heat is transferred more readily to the lower layers [43] and, smaller temperature differences occur throughout the soil profile. Smaller temperature differences will minimize the differences in the CO_2_ produced at the topmost soil layer compared with that produced at lower depths, thereby reducing lag times. Therefore, further investigations of the lag times between soil respiration and soil temperature under differences in soil water content are warranted.

## Conclusions

In this study, high-frequency soil respiration measurements were performed in a BSC-dominated ecosystem at the southeastern edge of the Tengger Desert in northern China to identify the temporal variations between soil temperature and soil moisture over the diel and seasonal cycles. Over the daily cycles, the proximate cause of the diel hysteresis between soil respiration rates and soil temperatures in the BSC-covered areas is likely a mismatch between the depth of the soil temperature and moisture measurements and the depth of CO_2_ production. Lag times between soil respiration rates and soil temperatures in the BSC-covered areas were negatively related to the soil moisture, which is possibly because water transfers heat more efficiently than air. This finding suggests that global carbon cycle models should account for the interactive effects of soil temperature and moisture on soil respiration in BSC-covered desert ecosystems. Moreover, the diel hysteresis phenomenon between soil respiration and soil temperature should be considered to accurately evaluate the total C budgets in BSC-dominated areas at the ecosystem level.

## Supporting information

S1 Table The minimum and maximum daily mean soil respiration for each month in the BSC-covered areas during the experimental periods from November 2015 to October 2016 in the Shapotou region of the Tengger Desert, northern China. (DOCX)
