# Supplementary material for: Diel hysteresis between soil respiration and soil temperature in a biological soil crust covered desert ecosystem
Source: PLoS One. 2018 Apr 6;13(4):e0195606. doi: 10.1371/journal.pone.0195606 (PMC5889175; doi:10.1371/journal.pone.0195606)
Supplement: S1 Table — (DOCX) [file pone.0195606.s001.docx]

**S1 Table. The minimum and maximum daily mean soil respiration for each month in the BSC-covered areas during the experimental periods from November 2015 to October 2016 in the Shapotou region of the Tengger Desert, northern China.**

| **Time** | **Crust type** | | | |
| --- | --- | --- | --- | --- |
|  | **moss-dominated crusts** | | **lichen-dominated crusts** | |
|  | **minimum value**  **(μmol m^-2^ s^-1^)** | **maximum value**  **(μmol m^-2^ s^-1^)** | **minimum value**  **(μmol m^-2^ s^-1^)** | **maximum value**  **(μmol m^-2^ s^-1^)** |
| **Nov 2015** | 0.063 ± 0.020 | 0.418 ± 0.017 | 0.023 ± 0.013 | 0.493 ± 0.043 |
| **Dec 2015** | 0.002 ±0.030 | 0.284 ± 0.026 | -0.020 ± 0.023 | 0.174 ± 0.016 |
| **Jun 2016** | -0.052 ± 0.025 | 0.079 ± 0.017 | -0.032 ± 0.010 | 0.062 ± 0.020 |
| **Feb 2016** | -0.020 ± 0.022 | 0.143 ± 0.018 | -0.010 ± 0.017 | 0.108 ± 0.012 |
| **Mar 2016** | 0.336 ± 0.019 | 0.942 ± 0.081 | 0.222 ± 0.036 | 1.149 ± 0.096 |
| **Apr 2016** | 0.093 ± 0.017 | 0.776 ± 0.078 | 0.009 ± 0.010 | 0.945 ± 0.092 |
| **May 2016** | 0.239 ± 0.015 | 1.547 ± 0.107 | 0.120 ± 0.015 | 1.600 ± 0.154 |
| **Jun 2016** | 0.272 ± 0.015 | 2.059 ± 0.245 | 0.223 ± 0.024 | 2.053 ± 0.229 |
| **Jul 2016** | 0.033 ± 0.023 | 3.329 ± 0.106 | 0.099 ± 0.027 | 3.514 ± 0.149 |
| **Aug 2016** | 0.193 ± 0.013 | 2.664 ± 0.094 | 0.233 ± 0.017 | 3.357 ± 0.129 |
| **Sep 2016** | 0.120 ± 0.017 | 1.242 ± 0.196 | 0.149 ± 0.034 | 1.917 ± 0.170 |
| **Oct 2016** | 0.038 ± 0.012 | 0.829 ± 0.042 | 0.086 ± 0.018 | 1.426 ± 0.123 |
